# Fixation failure in patients with traumatic diastasis of pubic symphysis: impact of loss of reduction on early functional outcomes

**DOI:** 10.1186/s13018-021-02802-x

**Published:** 2021-11-06

**Authors:** Claudio Rojas, Ernesto Ewertz, Jose Miguel Hormazábal

**Affiliations:** 1 Department of Orthopaedic Surgery, Hospital del Trabajador de Santiago, Ramón Carnicer 185, 7501239 Santiago, Chile; 2 Department of Orthopaedic Surgery, Clínica Dávila, Recoleta 464, 8431657 Santiago, Chile

**Keywords:** Symphyseal disruption, Pubic diastasis, Functional outcomes, Fixation failure, Loss of reduction, Majeed’s, APC pelvic fracture

## Abstract

**Background:**

Failure of fixation (FF) in pubic symphysis diastasis (SD) ranges between 12 and 75%, though whether it influences functional outcomes is still debated. The objective of this study is to evaluate the impact of anterior pelvic plate failure and loss of reduction on Majeed’s functional scores.

**Methods:**

Single center retrospective review of consecutive patients with acute SD treated by means of anterior pubic plating. Thirty-seven patients with a mean age 45.7 ± 14.4 years were included. Demographics, AO classification, pelvic fixation and secondary procedures were recorded. Majeed’s functional scores at minimum 6 months follow-up were compared according to the presence of FF and loss of reduction.

**Results:**

Fifteen patients presented FF. Eight presented an additional loss of symphyseal reduction. Mean Majeed´s score (MMS) in patients with and without FF was 64.4 ± 13.04 and 81.8 ± 15.65, respectively (*p* = 0.0012). Differences in MMS in patients without FF and those with FF and maintained or loss of anterior reduction were 11.3 [70.5 vs 81.8] (*p* = 0.092) and 22.7 [59.1 vs 81.8] (*p* = 0.001), respectively. Significant association of FF with AO classification was noted. (OR 12.6; *p* = 0.002).

**Conclusions:**

Differences in MMS in the analyzed groups suggest that loss of reduction might be more relevant than failure of the anterior osteosynthesis in functional outcomes.

## Introduction

The pubic symphysis is a fibrocartilaginous joint composed of four strong ligaments that allows discrete multidirectional movement [1]. High energy mechanisms are required to injure these ligaments, and damage to surrounding structures can result in relatively high rates of hemodynamic instability and mortality in the acute setting [2–5].

Diverse fixation methods have been used to restore and maintain symphyseal reduction, though the most frequently utilized are external fixators and pubic plates [6–10]. As ligamentous structures heal and normal joint motion resumes, rigid fixation methods can result in metalwork loosening, plate breakage or loss of anatomic reduction. Failure rates of pubic plates ranging from 12 to 75% and significant loss of symphyseal reduction in 7 to 88% have been previously reported [11–15]. Furthermore, early failures within the first 30 days after surgery can present in as many as 11.6% of APC pelvic fractures [15]. Despite this, only 1.6 to 9% of patients will eventually require secondary surgical procedures [12, 14–19]. Therefore, whether plate loosening, breakage or loss of symphyseal reduction have an impact on functional outcomes is still a matter of debate [11, 20–22]. Given the paucity of literature regarding the relevance of anterior metalwork failure and loss of reduction, we sought to determine their influence in clinical outcomes.

The objective of this study is to evaluate the impact of anterior pelvic plate failure and loss of reduction on early Majeed’s functional scores.

## Methods

We conducted a retrospective cohort review at a single level I trauma center, of consecutive patients admitted with the diagnosis of pelvic fracture, between January 2016 and December 2018. Patients with acute symphyseal diastasis that were surgically treated using 4 to 6 hole 4.5 anterior symphyseal plates (Matta Pelvic System®; Stryker, Mahwah, NJ, USA) were included. Patients with incomplete radiological studies or unregistered Majeed functional scores were excluded. Population data regarding demographics, mechanism of injury, associated injuries, 2018 AO pelvic fracture classification [23] and osteosynthesis characteristics were recorded using a predefined data gathering spreadsheet. Patients were followed for a minimum of 6 months. Majeed’s functional scores [24] at 6 months were recorded. Digital radiological images (AGFA Xero viewer 8.1.2 system) after surgery were evaluated by two trained team members. Fixation failure (FF) was defined as presence of plate or screw breakage and screw loosening. Additional loss of symphyseal reduction was defined as an increase ≥ 5 mm in the distance between pubic bones in radiological follow up.

Patients were categorized in three groups according to the presence of FF and loss of symphyseal reduction. The groups are as follows: Group A, patients without radiological failure, group B, patients with FF and maintained reduction and Group C, patients with FF and additional loss of symphyseal reduction. Majeed’s functional scores between groups were compared. The rates of FF and severity of pelvic injury were also analyzed.

Statistical analysis was performed using Stata Statistical Software (Release 13. College Station, TX: StataCorp LP). Data are presented in means, ranges, and standard deviation. Normal distribution of the population was determined using the Shapiro–Wilk test. Differences were analyzed using t-student and Fisher’s exact test. A 95% confidence interval was used, and statistical significance was set at *p* value < 0.05 with IC 95%.

## Results

After inclusion and exclusion criteria were applied, 37 patients were eligible for final analysis. Thirty male and seven female patients, with a mean age of 45.7 years (SD: ± 14.4, range: 20–77 years), were included. The mechanism of injury was motor vehicle accidents in 23, fall from height in 9 and crushing injuries in 6 patients. Anterior pubic plating fixation was performed as follows. Four hole plate vs six hole 4.5 plates were used in 20 vs 2 patients in group A, 6 vs 1 in group B and 6 vs 2 in group C. Additional sacroiliac screw fixation was performed in thirty-five (94.6%) patients.

No signs of FF were observed in 22 patients (Group A). Fifteen patients presented FF; 7 patients maintained postoperative symphysial reduction (Group B) and 8 patients presented an additional loss of the anterior reduction (Group C). No cases of plate breakage were recorded. Two patients presented early failures that required secondary interventions; a catastrophic failure 17 days after the initial surgery that was revised using a double plating fixation and one deep wound infection that required surgical debridement, metalwork removal and revised to an external fixator as definitive surgery. No late secondary surgeries were recorded.

Immediate postoperative symphyseal distance in groups A, B and C was 6.2 mm (SD 1.87, range 4–10), 7.71 mm (DE 2.13, range 4–11) and 7 mm (DE 2.61, range 2–11), respectively. After failure, group C presented a mean distance of 15.5 mm (DE 2.82, range 11–20). Mean time from surgery to loss of reduction was 5 ± 2.7 weeks (range, 1–8). Detailed information is presented in Table 1.

**Table 1:** Details of patients with failure of pubic fixation (Groups B + C)

Age	Sex	AO Classification	Majeed score	Anterior fixation			Posterior Fixation	
				Postoperative diastasis (mm)	Loss of reduction (mm)	Time to loss of reduction (weeks)	Nº Levels	Type (sacroiliac screws)
57	M	B2.2	52	11	–	–	1	1si
63	M	B2.3	68	9	–	–	1	1 si
55	M	B3.3	62	8	–	–	1	2 si
28	F	B3.3	84	4	–	–	1	1 si
51	M	B3.3	71	7	–	–	1	1 si*
49	M	C1	84	8	–	–	1	2 tsti
51	M	C1	73	7	–	–	2	2 si 1 tsti
61	M	B2.2	58	8	18	8	1	1 si
40	M	B2.3	64	5	11	8	–	–
53	M	B2.3	64	8	14	8	1	1 si
60	M	B3.3	43	8	14	4	1	1 si 1 tsti
67	M	C1	44	11	16	1	1	2 tsti*
55	M	C1	51	7	14	4	2	3 si 1 tsti
41	F	C1	78	7	20	5	1	1 tsti
20	F	C2	71	8	17	2	1	3 si

^*^ = secondary interventions

si = sacroiliac screw /

*tsi* Transiliac-Transsacral Screw

The mean Majeed score (MMS) according to AO classification was 79.48 ± 15.8 and 62.1 ± 12.96 for AO-B and AO-C fractures, respectively (*p* = 0.019). The MMS in groups A, B and C was 81.8 ± 15.65, 70.5 ± 11.4 and 59.1 ± 12.54, respectively. Combined MMS for Groups B and C (failure group) was 64.4 ± 13.04. Significant differences between Group A and failure group (B + C groups) were observed (*p* = 0.0012).

Failure group was further analyzed according to presence or absence of loss of reduction. No significant differences in MMS when comparing groups A and B were observed. (*p* = 0.092). However, significant differences were observed in MMS between Group A and C (*p* = 0.001). Moreover, patients from Group C presented a decreased MMS of 22.7. (Table 2).

**Table 2:** Mean majeeds functional scores

Group (n) = score	No fixation failure	Fixation failure	
MMS	A (22) = 81.8	B(7) = 70.5	*p* = 0.092
		C(8) = 59.1	*p* = 0.001

^*^MMS Mean Majeed`s Functional Score

*p* p value

Failure rates by severity of pelvic injury were analyzed. Five (20%) B type and 10 (77%) C type fractures presented FF. (OR 12.6; range, 2.4–64.2; *p* = 0.002) [2].

### Discussion

In our series, 15 (41%) patients presented failure of the anterior pelvic fixation, which is similar to other reports in the literature [11]. Additional loss of anterior reduction (7.75 mm ± 2.71) was observed in eight cases (19%) presenting as late as 8 weeks postoperatively. Despite this, only two patients required early secondary interventions and no patients required late plate removal due to symptoms associated with loosening.

Even though no patient-reported outcome measures have been developed for pelvic trauma; generic outcome instruments (SF-36) and disease specific instruments have been used to assess patient functional performance [25]. The Majeed score has been widely used in research concerning the quality of life of patients with pelvic injuries [24]. We investigated how implant failure and loss of reduction influence the patient’s functional scores. The impact of FF has been previously studied. Giannoudis et al. found no correlation of its occurrence with worse functional outcome [14]. On the other hand, Frietman et al. [25] retrospectively evaluated 37 patients treated with anterior pelvic plating and reported higher Majeed scores and similar SF-36 scores in patients with implant failure. Conversely, we observed significant differences in mean Majeed scores of patients with and without FF (*p* = 0.012). Moreover, additional loss of anterior reduction resulted in significant differences in mean Majeed Score (*p* = 0.001); meanwhile, no differences were observed in cases with failure and maintained reduction (*p* = 0.092). These findings suggest that loss of anatomic reduction might be more relevant than loosening or failure of the anterior construct.

In this series, patients with FF were 12.6 times more likely to belong to the C type fracture group. Posterior fixation has been proposed as a method of augmentation in fractures with some component of posterior ligamentous injury in order to reduce anterior implant failure [26]. Currently, B3 fractures and C type fractures are managed with posterior fixation, most frequently using sacroiliac screws, though failure rates remain high [3, 4, 15, 27, 28].

This study has many limitations arising from its retrospective nature, and functional outcomes scores were not recorded at the same point in time. Also, the influence of associated injuries on Majeed scores was not evaluated. The studied population is composed entirely of workers entitled to compensation benefits, making it difficult to compare with other patient groups.

Even though the severity of pelvic fracture and associated injuries are the main determinants of functional outcomes in patients with traumatic pubic diastasis; loss of anatomic reduction does result in poorer outcomes. The rates of FF and loss of reduction are higher in pelvic fractures with significant injury to posterior structures (AO B3—C type fractures) and posterior arch stabilization should be performed to address them, though they do not prevent failure of the anterior fixation. Augmentation of the anterior construct by means of double plating, cables, tight rope supplementation or the use of larger plates should be considered in order to reduce failure rates, loss of reduction and improve the functional outcome in this group of patients.

## Data Availability

Not applicable.

## Abbreviations

FF: Failure of fixation
SD: Pubic symphysis diastasis
MM: Mean Majeed score

## References

[1] Becker I, Woodley SJ, Stringer MD (2010). The adult human pubic symphysis: a systematic review. J Anat.

[2] Walheim G, Olerud S, Ribbe T. Mobility of the pubic symphysis. Measurements by an electromechanical method. Acta Orthop Scand. 1984;55(2):203–208. doi:10.3109/17453678408992338 10.3109/17453678408992338 6545936

[3] Walheim GG, Slevik G (1984). Mobility of the pubic symphysis measurements by an electromechanical method and a roentgen stereographammetric method. Clin Orthop Relat Res.

[4] Meissner A, Fell M, Wilk R, Boenick U, Rahmanzadeh R. [Biomechanics of the pubic symphysis. Which forces lead to mobility of the symphysis in physiological conditions?]. Unfallchirurg. 1996;99(6):415–21 8767137

[5] Burgess AR, Eastridge BJ, Young JWR (1990). Pelvic ring disruptions: effective classification system and treatment protocols. J Trauma - Inj Infect Crit Care.

[6] Simonian PT, Routt J (1997). Biomechanics of pelvic fixation. Orthop Clin North Am.

[7] Simonian PT, Routt MLJ, Harrington RM, Tencer AF (1994). Box plate fixation of the symphysis pubis: biomechanical evaluation of a new technique. J Orthop Trauma.

[8] Simonian PT, Routt MLJ, Harrington RM, Mayo KA, Tencer AF. Biomechanical simulation of the anteroposterior compression injury of the pelvis. An understanding of instability and fixation. Clin Orthop Relat Res. 1994;309:245–56. 7994968

[9] Simonian PT, Schwappach JR, Routt MLJ, Agnew SG, Harrington RM, Tencer AF (1996). Evaluation of new plate designs for symphysis pubis internal fixation. J Trauma.

[10] Sagi HC, Ordway NR, DiPasquale T (2004). Biomechanical analysis of fixation for vertically unstable sacroiliac dislocations with iliosacral screws and symphyseal plating. J Orthop Trauma.

[11] Morris SAC, Loveridge J, Smart DKA, Ward AJ, Chesser TJS. Is fixation failure after plate fixation of the symphysis pubis clinically important? Trauma. Clin Orthop Relat Res. 2012;470:2154–2160. 10.1007/s11999-012-2427-z. 10.1007/s11999-012-2427-z PMC3392398 22707071

[12] Sagi HC, Papp S (2008). Comparative radiographic and clinical outcome of two-hole and multi-hole symphyseal plating. J Orthop Trauma.

[13] Collinge C, Archdeacon MT, Dulaney-Cripe E, Moed BR. Radiographic changes of implant failure after plating for pubic symphysis diastasis: an underappreciated reality? Trauma. Clin Orthop Relat Res. 2012;470:2148–53. 10.1007/s11999-012-2340-5 10.1007/s11999-012-2340-5 PMC3392370 22552765

[14] Giannoudis PV, Chalidis BE, Roberts CS (2008). Internal fixation of traumatic diastasis of pubic symphysis: is plate removal essential?. Arch Orthop Trauma Surg.

[15] Eastman JG, Krieg JC, Routt MLC (2016). Early failure of symphysis pubis plating. Injury.

[16] Lange RH, Hansen ST. Pelvic ring disruptions with symphysis pubis diastasis. Indications, technique, and limitations of anterior internal fixation. Clin Orthop Relat Res. 1985;NO. 201:130–137. 10.1097/00003086-198512000-00021. 4064397

[17] Matta JM. Indications for anterior fixation of pelvic fractures. Clin Orthop Relat Res. 1996;88: 96. 10.1097/00003086-199608000-00011. 10.1097/00003086-199608000-00011 8769439

[18] Putnis SE, Pearce R, Wali UJ, Bircher MD, Rickman MS. Open reduction and internal fixation of a traumatic diastasis of the pubic symphysis: one-year radiological and functional outcomes. J Bone Jt Surg Ser B. 2011;93 B(1):78–84. 10.1302/0301-620X.93B1.23941. 10.1302/0301-620X.93B1.23941 21196548

[19] Webb LX, Gristina AG, Wilson JR, Rhyne AL, Meredith JH, Hansen ST (1988). Two-hole plate fixation for traumatic symphysis pubis diastasis. J Trauma Inj Infect Crit Care.

[20] Raman R, Roberts CS, Pape HC, Giannoudis PV (2005). Implant retention and removal after internal fixation of the symphysis pubis. Injury.

[21] Tornetta P, Templeman DC (2005). Expected outcomes after pelvic ring injury. Instr Course Lect.

[22] Tornetta P, Dickson K, Matta JM. Outcome of rotationally unstable pelvic ring injuries treated operatively. Clin Orthop Relat Res. 1996. 10.1097/00003086-199608000-00018. 10.1097/00003086-199608000-00018 8769446

[23] Meinberg EG, Agel J, Roberts CS, Karam MD, Kellam JF. Fracture and dislocation classification compendium. J Orthoped Trauma. 2018. 10.1097/BOT.0000000000001063 10.1097/BOT.0000000000001063 29256945

[24] Majeed SA (1989). Grading the outcome of pelvic fractures. J Bone Jt Surg Ser B.

[25] Frietman B, Verbeek J, Biert J, Frölke JP (2016). The effect of implant failure after symphyseal plating on functional outcome and general health. J Orthop Trauma.

[26] Avilucea FR, Whiting PS, Mir H (2016). Posterior fixation of APC-2 pelvic ring injuries decreases rates of anterior plate failure and malunion. J Bone Jt Surg Am.

[27] Kankanalu P, Orfanos G, Dwyer J, Lim J, Youssef B (2020). Can locking plate fixation of symphyseal disruptions allow early weight bearing?. Injury.

[28] Metz RM, Bledsoe JG, Moed BR (2018). Does posterior fixation of partially unstable open-book pelvic ring injuries decrease symphyseal plate failure? A biomechanical study. J Orthop Trauma.

